# Optical charge state control of spin defects in 4H-SiC

**DOI:** 10.1038/s41467-017-01993-4

**Published:** 2017-11-30

**Authors:** Gary Wolfowicz, Christopher P. Anderson, Andrew L. Yeats, Samuel J. Whiteley, Jens Niklas, Oleg G. Poluektov, F. Joseph Heremans, David D. Awschalom

**Affiliations:** 1 Institute for Molecular Engineering, University of Chicago, Chicago, IL 60637 USA; 2 WPI-Advanced Institute for Materials Research (WPI-AIMR), Tohoku University, Sendai, 980-8577 Japan; 3 Department of Physics, University of Chicago, Chicago, IL 60637 USA; 4 Institute for Molecular Engineering and Materials Science Division, Argonne National Laboratory, Lemont, IL 60439 USA; 5 Chemical Sciences and Engineering Division, Argonne National Laboratory, Lemont, IL 60439 USA

## Abstract

Defects in silicon carbide (SiC) have emerged as a favorable platform for optically active spin-based quantum technologies. Spin qubits exist in specific charge states of these defects, where the ability to control these states can provide enhanced spin-dependent readout and long-term charge stability. We investigate this charge state control for two major spin qubits in 4H-SiC, the divacancy and silicon vacancy, obtaining bidirectional optical charge conversion between the bright and dark states of these defects. We measure increased photoluminescence from divacancy ensembles by up to three orders of magnitude using near-ultraviolet excitation, depending on the substrate, and without degrading the electron spin coherence time. This charge conversion remains stable for hours at cryogenic temperatures, allowing spatial and persistent patterning of the charge state populations. We develop a comprehensive model of the defects and optical processes involved, offering a strong basis to improve material design and to develop quantum applications in SiC.

## Abstract (web summary)

Defects in silicon carbide represent a viable candidate for realization of spin qubits. Here, the authors show stable bidirectional charge state conversion for the silicon vacancy and divacancy, improving the photoluminescence intensity by up to three orders of magnitude with no effect on spin coherence.

## Introduction

Optically active color centers in wide bandgap semiconductors have shown considerable potential for a variety of spin-based quantum technologies, from quantum computing and quantum memories^1^ to nano-scale sensing^2–4^. Spin defects in silicon carbide (SiC) in particular combine the optical properties required for single-spin measurements^5–10^ with wafer-scale growth and silicon-like fabrication capabilities developed for high-power electronics. However, optimizing these systems for spin qubit applications requires an understanding of not only their spin and optical properties, as demonstrated in the negatively charged silicon vacancy $\left( {{\rm{V}}_{{\rm{Si}}}^ - } \right)$ ^5, 11^ and the neutral divacancy (VV^0^)^10, 12–14^, but also an understanding of their charge properties.

Impurities in SiC and their charge states have been investigated for conventional electronics applications, as they play an important role in transport properties and in carrier compensation. Most studies involve deep level transient spectroscopy (DLTS)^15, 16^, electron spin resonance (ESR)^17, 18^ and density functional theory (DFT)^19–21^ with a strong focus on the carbon vacancy (V_C_)^16, 22, 23^; fewer works have addressed V_Si_ and VV defects^24^. For the purpose of quantum information, it is desirable to understand the complete physics of the defects themselves, not just their influence on transport or other electrical characteristics of the substrate.

Here we investigate the effect of optical illumination on the stability of the relevant (optically bright) charge states of VV and V_Si_, the ability to control and convert these states between different charge levels, and the implications for quantum applications. We investigate these questions using a combination of techniques including photoluminescence (PL), optically detected magnetic resonance (ODMR) and electron spin resonance (ESR). The VV and V_Si_ charge states are both stabilized to the VV^0^ and ${\rm{V}}_{{\rm{Si}}}^ -$ states required to observe PL, whose intensity can be enhanced by up to three orders of magnitude depending on the material (local defect concentrations and Fermi level). For VV in particular, we observe bidirectional charge conversion between the neutral (bright qubit state) and a dark charge state using mainly near-ultraviolet (365–405 nm) and near-infrared (976 nm) light. This charge conversion is stable at cryogenic temperature and does not affect the ODMR contrast nor the electron spin coherence time, and can therefore be readily applied to increase PL emission from ensembles.

Charge state conversion can have multiple origins, including one-photon and two-photon ionization, free carrier recombination, and charge transfer between defects. In order to fully understand the involved processes, we measure the charge dynamics of VV, V_Si_ and nitrogen (N) under illumination, where N is the main dopant in our semi-insulating 4H-SiC samples. Excitation dependence with wavelengths ranging from 365 to 1310 nm were measured and simulated, offering a comprehensive picture of charge transfer between these defects. In particular, this allows us to identify VV^−^ as being the possible dark charge state of the divacancy, while ${\rm{V}}_{{\rm{Si}}}^ -$ most likely converts to the dark ${\rm{V}}_{{\rm{Si}}}^0$ charge state with above-bandgap light.

Control and understanding of these charge dynamics is crucial for maximizing spin qubit readout, choosing adequate background impurity concentrations in samples and optimizing designs of SiC nano-devices for quantum applications. Such methods have also been applied in the nitrogen-vacancy (NV) center in diamond for quantum optics applications^25^, enabling for example reduced spectral diffusion^26^ or Stark tuning of the optical transitions through photo excitation of trapped charges^27^. More exotic applications of charge dynamics include high density data storage^28^, STED super-resolution imaging^29, 30^ and charge quantum buses^31^.

## Results

### PL enhancement of VV^0^ using UV illumination

**Fig. 1:**
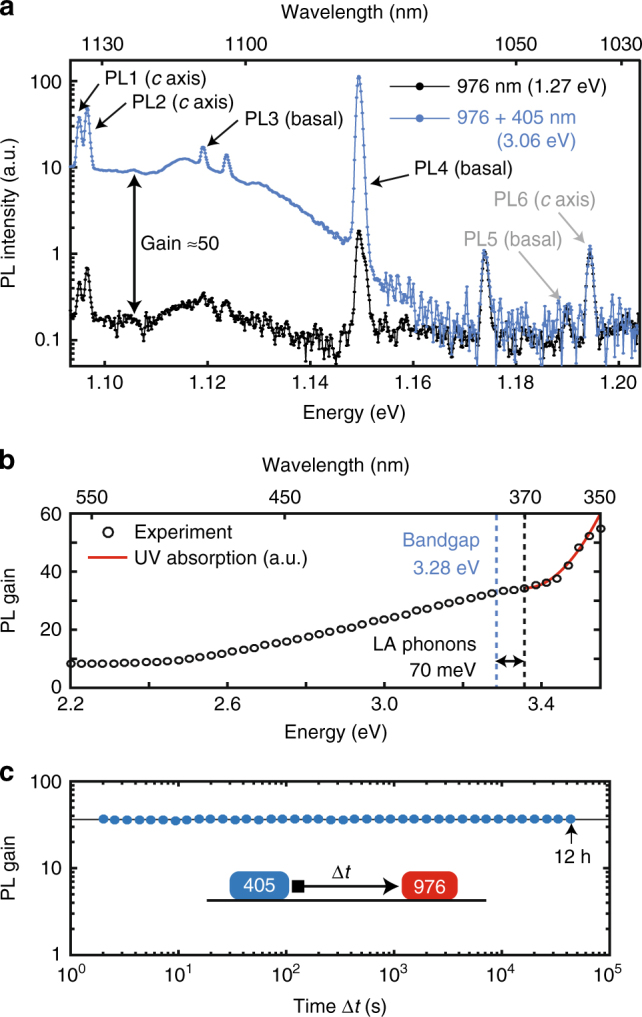
Effect of near-bandgap illumination on 4H-SiC divacancies. **a** PL spectrum with 976 nm excitation of the various divacancies in 4H-SiC, as designated in ref. ^6^, without and with continuous illumination at 405 nm (≈5 mW optical power). All the observed PL lines except PL5 and PL6 are enhanced by the UV excitation, including both *c* axis and basal defects. **b** Gain in the PL signal (integrated across PL1–4) as a function of excitation wavelength (energy) around the 4H-SiC bandgap (3.28 eV at 5 K^49^). Power was normalized to 0.4 μW across the entire energy range. The onset of change in the curve is shifted from the bandgap energy due to absorption of longitudinal acoustic phonons (about 70–80 meV). The UV absorption and corresponding electron-hole generation rate follows the curve in red, as given in ref. ^49^. **c** Lifetime of the VV^0^ charge state after a 405 nm pulse at 6 K. No significant decay (standard deviation of signal is 2%) is observed after 12 h

We initially observe a drastic increase in PL intensity of VV^0^, by about 50 times at 6 K, when continuously illuminating a semi-insulating 4H-SiC sample with a 405 nm (“UV”) laser diode, in addition to the 976 nm (1.27 eV) laser required for PL excitation. This is shown in Fig. 1a where the full PL spectrum for all the divacancies (PL1−PL6^6^) is taken with (blue) and without (black) 405 nm (3.06 eV) excitation. Both *c* axis (PL1, PL2) and basal defects (PL3, PL4) show an increase in their PL intensity, with slight variation between defects, which we ascribe to charge conversion of the divacancy toward its observable neutral state. On the other hand, PL5 and PL6 remain completely unaffected, adding another unique feature to these currently unidentified defects on top of their strong room temperature PL emission. The VV^0^ PL enhancement with UV was observed in all semi-insulating wafers we measured, with gains ranging by a factor of 2−1000 (Supplementary Fig. 2), including samples obtained from separate commercial suppliers (Cree or Norstel), different growth batches, or even simply from separate positions within the same wafer. This strongly indicates an influence from the local environment, e.g., from the remaining concentration of N dopants or other impurities which is known to locally differ in as-grown wafers^32^. The PL intensity with UV remains fairly constant however from sample to sample.

In order to understand the effect of 405 nm illumination, and optimize the enhancement, the excitation wavelength is swept across the 4H-SiC bandgap energy (3.28 eV, 380 nm) as shown in Fig. 1b. The PL gain slowly increases with excitation energy, and around 3.33–3.35 eV, slightly above the 4H-SiC bandgap (3.28 eV), it drastically turns up. This suggests two separate processes are altering the VV charge state from either VV^−^ or VV^+^ to VV^0^: at low energies, we will see this is due to direct photoionization, while at high energies, the gain results from capture of photo-generated carriers.

We now consider charge dynamics under illumination, starting from the stability of the conversion observed after UV excitation. As illustrated in Fig. 1c, the system is initially pumped with 405 nm toward a high VV^0^ population (strong PL intensity), followed by a long delay to allow for relaxation and finally measurement using 976 nm. No change is observed over the course of 12 h, a result largely expected for a deep defect at cryogenic temperature (6 K). More interestingly, the PL intensity always drops to a low level after turning off the UV excitation while 976 nm was continuously on. Combined with this long stability, this implies that the use of 976 nm to excite VV PL is simultaneously converting the VV out of its neutral-charge state, toward a dark state (more details are given later on). This has significant consequences as wavelengths near 976 nm have been extensively used in recent PL- and ODMR-related works^8, 33, 34^, owing to being close to the absorption maximum of the ground to excited state transition of VV^0^, as well as being easily available commercially. These previous studies may therefore have been partially perturbed by charge conversion.

### Illumination effects on VV^0^ spin properties

**Fig. 2:**
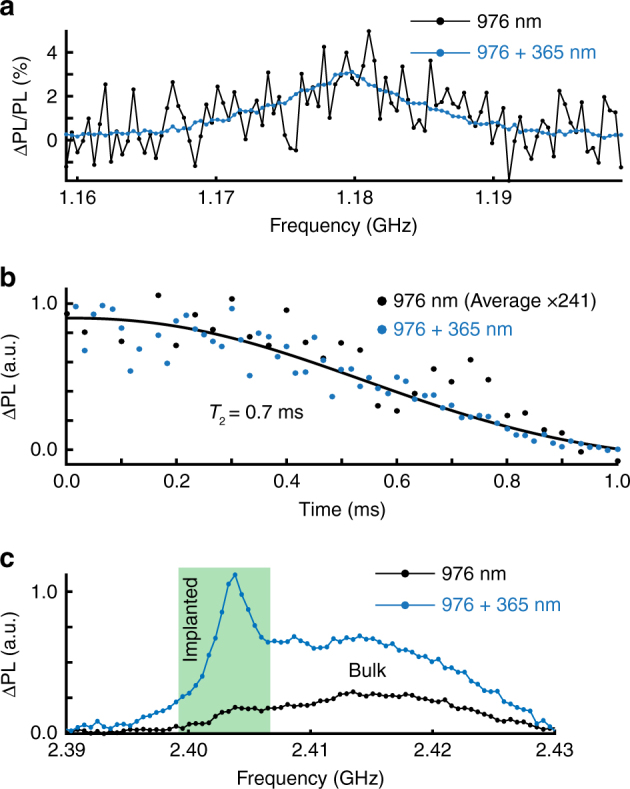
Charge conversion effect on the spin properties. ODMR signals are given as relative photoluminescence intensities (ΔPL) under microwave excitation. **a** CW-ODMR spectrum at 50 G and measured through a monochromator at the 1130.6 nm PL2 zero-phonon line to ensure no other contribution in the optical signal. The intensity is given as the ratio (i.e., contrast) between the ODMR and PL intensity, which remains constant with and without 365 nm illumination, indicating unchanged spin polarization and readout mechanisms. **b** Hahn echo decay experiment for PL2 at ≈400 G, measured with pulsed-ODMR at 6 K. The 365 nm excitation is continuous throughout the sequence, resulting in a signal increase while the coherence time is unaffected. Decay with 976 nm excitation only was averaged 241 times more than the decay with also 365 nm illumination. Line (in black) is a stretched exponential fit (stretch factor ≈2) to the data. **c** CW-ODMR (PL2 at ≈400 G) of a 4H-SiC sample with a 500 nm carbon-implant layer below the surface. The divacancies created at the layer are barely visible before 365 nm excitation. The implanted layer peak is also shifted from the bulk due to a magnetic field gradient across the sample

Above-bandgap excitation can be used to efficiently convert VV toward its neutral-charge state, and more importantly drastically increases the PL intensity. For practical applications, however, we verify this has no effect on the spin properties of VV^0^. In Fig. 2a, we first measure the ODMR contrast of PL2 at 50 G, i.e., the ratio of ODMR over PL intensity, which provides a direct measure of how the spin states may be affected during illumination. For these experiments, the 405 nm laser is replaced by a 365 nm (also called “UV”) light-emitting diode which is more efficient at charge conversion since it is above bandgap in energy. No difference in contrast is observed with or without 365 nm, and the charge conversion therefore does not significantly affect the spin state nor the readout mechanism. However, the signal-to-noise ratio improves by ~70 times with illumination due to increased VV^0^ charge population. More details are given in the Supplementary Fig. 6, Supplementary Note 2 regarding the ODMR experiments presented here.

A second crucial property is the electron spin coherence of the defect. From Fig. 1c, the charge stability at cryogenic temperature (6 K) is shown to be much longer than any coherence timescale^34^; however, we check that even with constant 365 nm illumination (~0.2 mW) and corresponding electron-hole pair generation, the coherence time remains unaffected. At 400 G, the ensemble electron spin coherence *T*_2_ is measured to be 0.7 ms and remains completely unaffected by either light or free carriers (Fig. 2b), while much longer averaging (>×200) was required to obtain similar signal-to-noise ratios without 365 nm illumination. This is not an obvious result as scattering or exchange interaction with free carriers can easily reduce *T*_1_ or *T*_2_ of defect spins^35^.

Until now, all measurements were realized on as-grown commercial wafers with naturally occurring impurity concentrations. However, carbon ion implantation or electron irradiation^14^ is often used to increase the PL intensity and to improve spatial resolution. The type of defects created by lattice damage during these processes cannot be well controlled, though it may be partially managed by annealing, and the local Fermi level may shift away from the desired charge state. We test this with a 500 nm thick layer of implanted divacancies (Methods section). When measuring the PL2 ODMR spectrum of this sample (at ≈400 G), as shown in Fig. 2c, we obtain a broad “bulk” signal observable across the entire sample depth using simply 976 nm excitation. When 365 nm is turned on (with constant absorption over the sample depth), the bulk intensity increases as expected, but more importantly a narrower and more intense peak appears. The latter is assigned to the implanted layer which, being confined in depth, is less sensitive to inhomogeneity in the static magnetic field. Rabi experiments at the peak layer frequency yielded as expected an increased contrast (Supplementary Fig. 7), demonstrating that the UV charge stabilization can be critical in such samples.

### VV charge state conversion

**Fig. 3:**
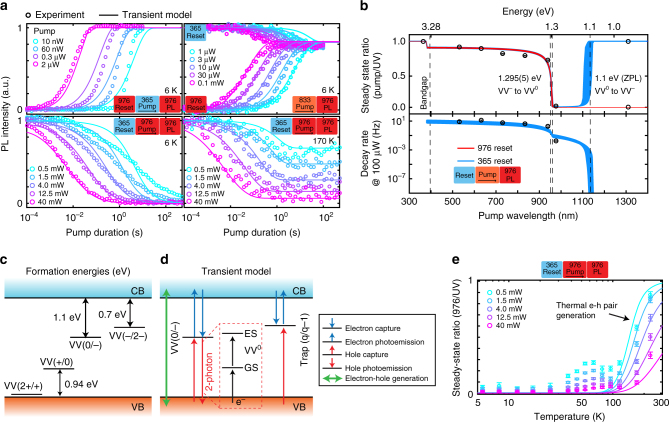
Photo-dynamics and modeling in neutral divacancies in 4H-SiC. The charge dynamics is probed using two and three color experiments, following a reset-pump-measure scheme. **a** Typical decay curves obtained under various reset/pump wavelength and temperatures. The fit (line) is obtained from the model given in **d**. **b** Top figure: ratio between pump and 365 nm steady-state PL intensities. Bottom figure: decay rates (normalized to 100 μW at every pump wavelength) obtained by fitting the decays in **a** with a stretched exponential function (error bars are 95% confidence intervals from the fit). In blue, the sequence starts after 365 nm pumping, while in red, after 976 nm. The lines are given by the model in **d**, with the area corresponding to 95% confidence intervals. For 1310 nm, no significant decay was observed over 100 s, hence the steady-state values are given without error bars. **c** Formation energies of the divacancy in 4H-SiC, taken from^20^. **d** Model used for simulating all transients in **a**, **b**, **e**, including the VV^0^ and VV^−^ levels of the divacancy, as well as an unknown trap with two charge states. Processes included in the model are given in the legend. Hole photo-emission converting VV^0^ to VV^−^ involves a two-photon process, exciting VV^0^ from its ground state to its excited state, followed by excitation and capture of an electron from the valence band. **e** Temperature dependence of the steady state after 976 nm pumping (365 nm reset). Error bars are 95% confidence intervals from the decays’ stretched exponential fit. Lines are given by the model in **d**, corresponding to thermal generation of electron-hole (e-h) pairs. The origin of the intermediate region between 30 and 100 K is unknown. Above 210 K, PL5 and PL6 signals become dominant, making the measurement unreliable as they are UV-insensitive

We now consider in more depth the charge mechanisms within 4H-SiC, in particular the effect of 976 nm illumination which appears to convert VV toward a dark charge state (VV^−^ or VV^+^). Since 976 nm is used both for PL excitation and causes charge conversion, it is necessary to separate the two contributions from the PL intensity, which can be achieved by looking at the conversion dynamics under pulsed light. This is realized using a three-pulse scheme: reset with either 976 or 365 nm, pump with a wavelength ranging from 365 to 1310 nm using various laser diodes, and measurement with 976 nm. Typical decay curves as a function of pumping duration are shown in Fig. 3a with different initial reset lasers, pump wavelength, pump power and temperatures.

In Fig. 3b, fitted steady states and decay rates (see Methods section regarding the fitting) are plotted as a function of pump excitation wavelength. Steady-state intensities are all normalized by the steady-state PL intensity after UV pumping. A clear transition is observed between 940 and 976 nm, at about 1.3 eV, for both steady-state values and decay rates, with shorter wavelengths being increasingly more efficient at charge conversion toward VV^0^. In addition, we measure a single wavelength at 1310 nm (0.95 eV) that tentatively suggests a second transition (between 976 and 1310 nm), where the VV^0^ charge state becomes insensitive to excitation (no observed decay).

The wavelength transitions can be related to photoionization energies and, though the Franck−Condon shift is unknown here, to formation energies obtained from DFT calculations^20, 36^ and reproduced in Fig. 3c. The divacancy defect in 4H-SiC has four stable charge states: +, 0, − and 2−. The (+/0) and (0/−) transition levels are calculated to be, respectively, ~*E*_v_ + 1 eV and ~*E*_c_ − 1.1 eV, with *E*_v_ and *E*_c_ the valence and conduction band energies. On the one hand, these energies correspond closely to ionization from either VV^+^ or VV^−^ (dark) to VV^0^ (bright), and therefore to the observed 1.3 eV optical transition. On the other hand, the minimum energy to photoionize VV^0^ to the dark state will be around 2 eV, requiring a two-photon absorption mechanism using 976 nm illumination. VV^0^ can absorb one photon to reach its excited state followed by a second photon to ionize either to VV^+^ or to VV^−^ (Supplementary Fig. 5). As two-photon absorption requires at least the ZPL energy (~1.1 eV, PL1−PL4) to reach the excited state, we expect no decay from VV^0^ should occur below this energy as was observed using 1310 nm illumination.

**Fig. 4:**
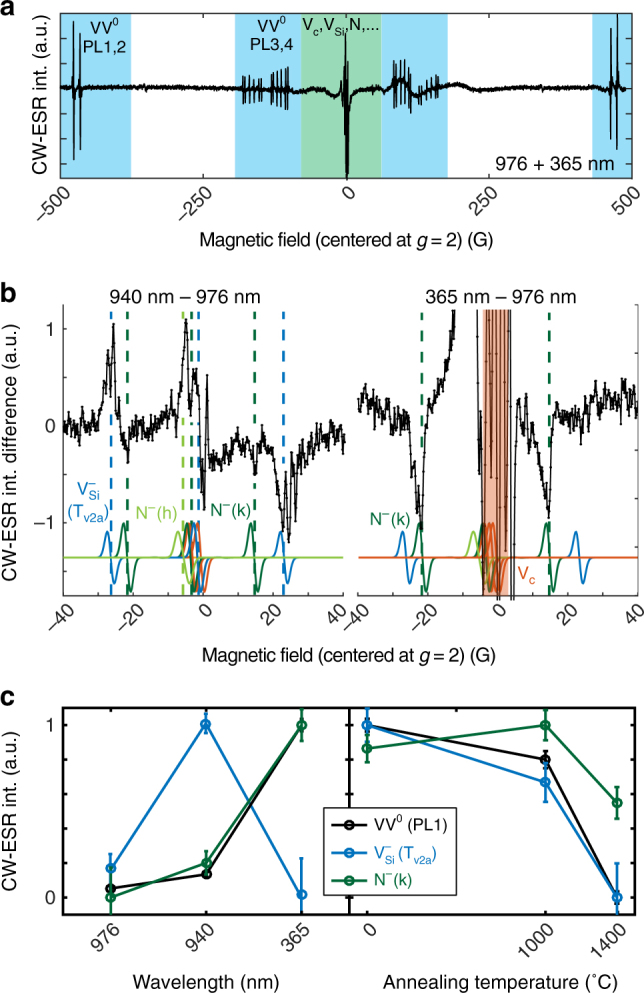
ESR at 15 K in semi-insulating 4H-SiC under illumination. **a** CW-ESR spectrum measured at 9.7 GHz, and centered around *g* ≈ 2 (≈3470 G, aligned to the *c* axis). VV PL1–4 are highlighted in blue, while defects such as N, V_C_, or V_Si_ are close to *g* ≈ 2 and highlighted in green. **b** Differential CW-ESR spectrum between either 940 nm and 976 nm excitation (left), or between 365 and 976 nm (right). Gaussian derivative lineshapes are simulated in color for known defects in 4H-SiC^18^. Their amplitudes only take into account transition probabilities, and not spin polarization or microwave saturation. **c** Normalized (per defect) CW-ESR intensity under 976, 940, and 365 nm (left) and for different annealing condition of the sample (right). For VV^0^, 365 nm is combined with 976 nm for spin polarization (and obtain enough signal). For the annealing dependence, the intensity was fitted under the best illumination condition for each defect, that is the maximum signal in the left. Annealed samples were only used in this panel (**c**, right). Error bars are 95% confidence intervals from the fit

Both VV^−^ and VV^+^ are candidates for the dark VV charge state, however, VV^−^ is more likely from our results. Our semi-insulating samples are compensated N-type samples with nitrogen (N) as primary dopant and carbon vacancies (V_C_) for compensation. Both species have states lying close to the conduction band (Supplementary Fig. 4). After UV illumination, free electrons will easily be recaptured by N (Fig. 4c) and possibly V_C_, allowing free holes to convert VV from VV^−^ to VV^0^. In the following sections we assume that VV^−^ is the dark state, though additional studies will be needed to fully resolve this question. It should be mentioned that during review of this paper we became aware of a similar work^37^ on optical charge conversion of divacancies in 4H-SiC that hypothesizes VV^+^ instead of VV^−^.

We attempt to model the observed dynamics using the rate-equation model shown in Fig. 3d, based on charge transfer between the divacancy and a trap of unknown origin. More details are given in both the Methods section and Supplementary Note 1. Simulated decays and their corresponding rates and steady states are shown in Fig. 3a, b. Three experimental characteristics are nicely reproduced by the model here: (i) the jump in charge conversion efficiency for above bandgap illumination, (ii) the VV^−^ to VV^0^ transition fitted to be 1.295(5) eV, and (iii) the VV^0^ to VV^−^ transition set at the ZPL energy (1.1 eV).

Finally, a temperature dependence of 976 nm pumping (365 nm reset) is taken between 5.5 and 210 K, with corresponding steady-states value shown in Fig. 3e. Above 100 K, the effect of 976 nm pumping compared to 365 nm pumping is drastically reduced. The simulation is able to reproduce this feature owing to thermal electron-hole generation, with possible origins being the thermal emission of shallow defects, such as nitrogen (≈0.05–0.1 eV) and boron (≈0.3 eV), capture barriers or changes in carrier lifetimes. From the simulation fit, this process has a characteristic activation energy around 0.1–0.2 eV depending on the fit dataset.

In summary, we identify a sharp transition of the VV charge dynamics at around 1.3 eV (960 nm), likely corresponding to the ionization of VV^−^ to VV^0^. Below 1.3 eV in energy, two-photon absorption drives the charge state toward VV^−^, while above, VV is preferentially in VV^0^ and remains stable for many hours after illumination. In addition, UV light above bandgap strongly drives the system toward VV^0^.

### Charge transfer between major defects

The experiments described previously made use of PL as a direct measurement of the divacancy neutral-charge state, combined with photo excitation to probe relevant energy levels as well as trapping or recombination dynamics. However, understanding all the major defects in 4H-SiC, not just the divacancy, is required to obtain a comprehensive picture of the sample behavior under illumination. While PL of V_Si_ can be measured, other important spin impurities, such as V_C_ or N are not photo-active, with no optical excited states in the bandgap. We thus turn toward electron spin resonance (ESR) to provide information on all the spin species.

A CW-ESR spectrum at X-band is shown in Fig. 4a with resonance peaks from PL1 to PL4 VV defect types, as well as a cluster of signals near the g-factor *g* = 2, known to be from V_Si_, V_C_ and/or N^13, 18^. In order to properly resolve some of these peaks, we subtract the ESR spectrum measured with 976 nm illumination from the spectrum obtained with either 940 or 365 nm illumination. The differential spectra, shown in Fig. 4b, then correspond to possible charge transfer with VV which is extremely sensitive to these wavelengths (further considerations are discussed in Methods section). The ESR peak intensities are given for the main identified defects in Fig. 4c (left). With 940 nm, two sets of resonances can be clearly assigned, the strongest due to ${\rm{V}}_{{\rm{Si}}}^ -$ (*T*_*V*2*a*_ or *V*2 center) and the weakest from N^−^ (k site). At this wavelength, VV^−^ undergoes photoionization to become VV^0^, emitting an electron to the conduction band which is likely captured by ${\rm{V}}_{{\rm{Si}}}^0$, and resulting in an increase in ${\rm{V}}_{{\rm{Si}}}^ -$. With 365 nm, large changes can be seen around *g* = 2, possibly from free electrons and V_C_, though the peaks are too clustered to be resolved. On the side of *g* = 2, N^−^ appears much stronger while the ${\rm{V}}_{{\rm{Si}}}^ -$ peaks completely disappeared. While this may be due to carrier-induced spin relaxation, such behavior is also well explained by charge dynamics: N, initially in its neutral-charge state due to either photoionization or thermal emission (shallow donor) before cooling down the sample, captures most of the generated electrons to give a high N^−^ signal. The holes now in majority, are captured by the various deep defects, with VV^−^ being converted toward VV^0^ (high signal), ${\rm{V}}_{{\rm{Si}}}^ -$ toward ${\rm{V}}_{{\rm{Si}}}^0$ (low signal), and possibly ${\rm{V}}_{\rm{C}}^{\rm{0}}$ toward ${\rm{V}}_{\rm{C}}^ +$ (high signal).

ESR measurements are often used to characterize the optimal annealing temperature for sample preparation. Changes in ESR intensity after sample annealing normally indicates variations in defect concentration, but can also be confused with a shift in the local Fermi level. After charge conversion, this second explanation is much less plausible. In Fig. 4c, the ESR intensity under best illumination condition (highest signal for each defect) was tracked for N, V_Si_, and VV for different annealing temperatures. Between 1000 and 1400 °C, the ESR signal of V_Si_ and VV significantly drops, which can be related to the defects becoming mobile followed by creation of multi-vacancies such as V_C_−V_Si_−V_C_^38–41^.

**Fig. 5:**
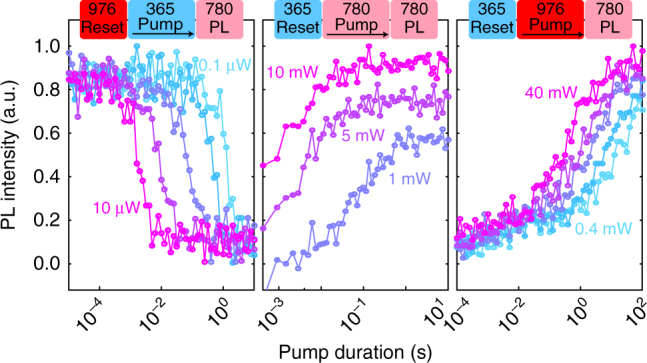
Photo-dynamics of ${\rm{V}}_{{\rm{Si}}}^ -$ at 6 K. Reset-pump-measure scheme similar to Fig. 3, but with 780 nm to excite PL in ${\rm{V}}_{{\rm{Si}}}^ -$ instead of 976 nm for VV^0^. Each of the curves correspond to different pump powers (logarithmic increment for 365 and 976 nm). 365 nm reduces the PL intensity, likely from charge conversion to ${\rm{V}}_{{\rm{Si}}}^0$. Both 976 and 940 nm reinitialize VV toward its bright ${\rm{V}}_{{\rm{Si}}}^ -$ state, but with different rates and power dependence, indicating charge transfer with VV. Charge conversion was measured to be persistent without light on the experiment timescales

The ESR experiments indicate a strong relationship between VV and V_Si_, as they are both affected by the 940–976 nm transition and by similar annealing temperatures. With ${\rm{V}}_{{\rm{Si}}}^ -$ being also a photo-active qubit of interest, we directly measure its PL by exciting the sample with 780 nm (Supplementary Fig. 1)^5, 42^. Three-pulse experiments for ${\rm{V}}_{{\rm{Si}}}^ -$ are shown in Fig. 5 with pumping using 365, 976 nm, as well as 780 nm as a replacement for 940 nm which was impractical here. 365 nm pumping drastically decreases the ${\rm{V}}_{{\rm{Si}}}^ -$ intensity while both 976 and 780 nm convert back the charge state to ${\rm{V}}_{{\rm{Si}}}^ -$.

These observations are consistent with the ESR experiments. Under 976 nm illumination, the V_Si_ PL signal recovers very slowly, with corresponding low ESR signal, and with little power dependence indicating competition between different carrier capture processes. In this case, VV^0^ emits holes by inefficient two-photon absorption, while N (and likely V_C_) emits electrons which will dominantly convert ${\rm{V}}_{{\rm{Si}}}^0$ toward ${\rm{V}}_{{\rm{Si}}}^ -$ after capture. At 940 nm, VV^−^ also now emits electrons allowing for faster conversion to ${\rm{V}}_{{\rm{Si}}}^ -$. At 780 nm, it is likely that conversion via capture is enhanced by direct photoionization of ${\rm{V}}_{{\rm{Si}}}^{\rm{0}}$ with (0/−) transition energy at *E*_v_ +1.3–1.5 eV (^20, 43^, reproduced in Supplementary Fig. 4). For completeness, the V_Si_ (−/2−) transition with a formation energy of *E*_c_ −0.6–0.8 eV is likely also excited at any wavelength below 976 nm, and therefore V_Si_ is more likely to be trapped in ${\rm{V}}_{{\rm{Si}}}^ -$ than in the 2− charge state. 780 nm illumination is therefore suitable for both ${\rm{V}}_{{\rm{Si}}}^ -$ PL excitation and ${\rm{V}}_{{\rm{Si}}}^ -$ charge stabilization.

**Fig. 6:**
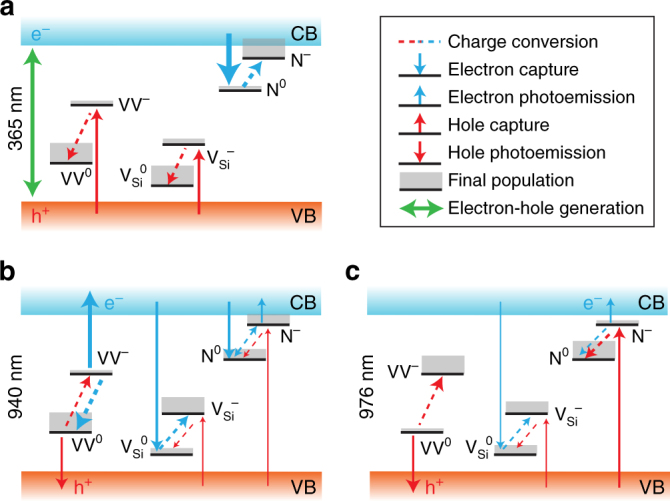
Summary of charge transfer in semi-insulating 4H-SiC under various illumination conditions. Strong transitions are shown by thicker arrows, and the steady-state population after illumination is approximatively represented by the gray area over each state. **a** Above-bandgap excitation and electron-hole generation. **b** Excitation above the VV^−^ photoionization transition (~1.3 eV). **c** Excitation below the VV^−^ photoionization transition, but above VV^0^ two-photon absorption

The full VV/ V_Si_ /N charge conversion picture under illumination is finally summarized in Fig. 6 for the three critical wavelengths explored in this work: 976, 940, and 365 nm. V_C_ was not taken into account due to lack of experimental measurements.

### Summary of charge dynamics

Our overall summary of the charge conversion is as follows: (i) Under 365 nm excitation and electron-hole generation, N dominantly traps electrons toward N^−^, while VV and V_Si_ capture the remaining holes to become VV^0^ and ${\rm{V}}_{{\rm{Si}}}^{\rm{0}}$. (ii) Below but close to 940 nm in wavelength, VV chiefly emits electrons and ends up in VV^0^, while V_Si_ captures those electrons to become ${\rm{V}}_{{\rm{Si}}}^ -$. N will be in an intermediate charge state as it absorbs both electron and holes, as well as being photoionized. (iii) At wavelengths higher than 976 nm, VV is converted to VV^−^ by slow hole emission to the valence band through a two-photon process; N is still photoionized resulting in N^0^ while V_Si_ capture both holes and electrons for a slow conversion toward ${\rm{V}}_{{\rm{Si}}}^ -$.

The charge conversion and transfer mechanisms presented throughout this work should remain valid in most semi-insulating materials, where defects are in comparable concentrations. For n- or p-doped materials, impurities can of course still be photoionized, however, electron-hole generation with above-bandgap light will likely set the local Fermi level to a different equilibrium than what is seen here.

### Toward applications in charge patterning

To complete this study, we turn toward applications using our ability to control the VV charge state. In recent experiments in diamond^28^, optical conversion between the NV^−^ and NV^0^ states was used to demonstrate the possibility of information storage by 3D patterning of the charge state. Because data can be both encoded in 3D, as well as a gradient of charge conversion, high storage densities can theoretically be achieved. We present a similar demonstration of charge patterning in 4H-SiC, and though our experiments are realized at 6 K, offer the potential for storage across entire wafers compared to diamond. The VV charge conversion works relatively well up to 150–200 K, and may possibly be extended to room temperature with the adequate choice of material (dominant dopant or impurity concentration).

**Fig. 7:**
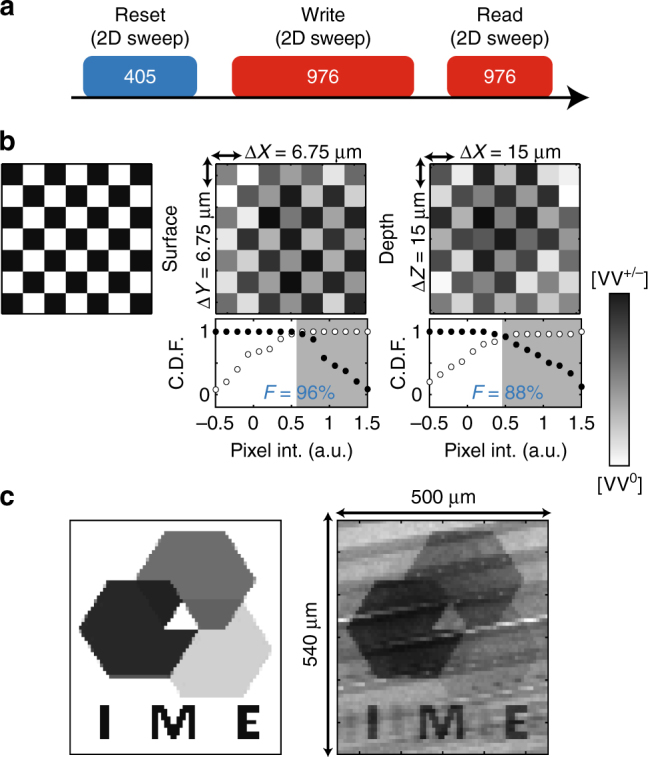
Spatial and amplitude control of the divacancy charge conversion. **a** Imaging sequence for **b**, **c**, with three sequential 2D sweeps: (1) Reset to high VV^0^ concentration using 405 nm. (2) Write using 976 nm with varying duration for charge conversion back to a desired lower VV^0^ concentration. (3) Read with a fast 976 nm pulse. **b** Pixel test of spatial control with, from left to right, the original pattern, the measured pattern in the *X*−*Y* plane (parallel to the sample surface) and the measured pattern in the *X*−*Z* plane (orthogonal to the sample surface). For *X*−*Z*, the intensity is normalized to the PL collection efficiency across the sample depth. Below each image, the corresponding cumulative distribution function (C.D.F.) is plotted from cumulative binning of the pixels according to their intensity (int.) and expected color (black/white dots for black/white pixels). The fidelity F is defined as $1 - P\left( {{\rm{b}}\left| {\rm{w}} \right.} \right) - P\left( {{\rm{w}}\left| {\rm{b}} \right.} \right),$ where $P\left( {i\left| j \right.} \right)$ is the probability of measuring *i* expecting *j*, where *i*, *j* = black (b), white (w), and the expected color is given by the background color in the plot^50^. **c** Amplitude control of the charge conversion using a gray scale image (left: original image, right: experiment)

The patterning scheme is presented in Fig. 7a with: a UV (405 nm) pulse to initialize the sample toward VV^0^, a write pulse with 976 nm to selectively obtain VV^−^ (or possibly VV^+^), and finally a short read pulse using 976 nm. The measurement pulse here weakly erases the information due to undesired charge conversion, which is the main limitation to this technique. In Fig. 7b, we test the spatial resolution of our setup by patterning a checkerboard design (left) where each square is a single pixel. This is realized first parallel to the sample plane (middle) and then in depth, orthogonal to the sample plane (right). Finally, for each pixel, we allow control over the amount of charge conversion, increasing the density of information that can be locally stored. This is demonstrated in Fig. 7c by patterning a 500× 540 μm gray scale image parallel to the sample surface.

## Discussion

In this work, we have systematically investigated the charge properties of divacancies in semi-insulating 4H-SiC, as well as other relevant defects, such as V_Si_ and N. Through optical excitation with wavelengths spanning from 365 to 1310 nm, VV was found to be stable in either its bright (neutral) or dark (negative or positive) charge configuration. The photoionization energy required to obtain the bright VV^0^ charge state was fitted to be around 1.3 eV, while below in energy two-photon ionization most likely converts VV to VV^−^. As a consequence, the commonly used 976 nm (below 1.3 eV) excitation is found to be detrimental for PL measurements. Considering the maximum of the phonon sideband of VV^0^, an excitation wavelength around 900–940 nm would be optimal, with commercial lasers readily available in this region. Above-bandgap excitation efficiently reshuffles the charge states of all defects, turning VV bright (VV^0^) and V_Si_ dark (possibly ${\rm{V}}_{{\rm{Si}}}^0$).

While all the results here were realized in ensembles of defects, they should be equally applicable to single defects in similar SiC materials. A possible major difference may arise from the usually lower background impurity concentration required for single defect samples, which would change the charge dynamics and optical conversion. Similar to the NV center in diamond^25, 44^, the appropriate wavelength could reduce blinking due to transient charge conversion of single defects and increase their photoluminescence count rate.

Overall, taking into account multiple impurities was necessary to obtain a complete picture of charge effects in the SiC samples; such considerations are crucial for tuning wafer growth techniques, samples with implanted layers, surface impurities or for devices with complex electric potentials. Finally, we confirmed that these optical charge conversions drastically improve the PL intensity and do not measurably impact the spin properties (ODMR, coherence). Combined with recent studies^10, 45^ characterizing the spin and optical properties of VV or V_Si_ in 4H and 3C-SiC, this work on charge conversion/stabilization helps to complete the suite of techniques and technologies realized in NV centers in diamond for use in SiC, while allowing for novel applications such as optically controlling the charge of spins in electronic devices realized in SiC. This study will also be relevant to spin-to-charge conversion in SiC, though further work is necessary.

## Methods

### Samples

All measurements were performed on commercially available high-purity semi-insulating 4H-SiC diced wafers purchased from Cree^32^, and using a scanning ODMR microscopy setup. Similar wafers have been used in other studies, with measured defect concentrations of N, VV, V_Si_ all in the order of 10^14^–10^16^ cm^−3^ ^32, 46, 47^. For the implanted sample, a high energy carbon implant ([^12^C] = 10^13^ cm^−2^,190 keV, 900 °C anneal for 40 min) was used, resulting in a calculated (SRIM software) 500 nm thick layer of divacancies. For ODMR, the samples are fixed to a printed circuit board patterned with a coplanar waveguide for magnetic resonance, and mounted in a closed-cycle cryostat cooled down to 5–6 K (unless otherwise mentioned). PL, ODMR, and ESR experiments were all realized on ensembles of defects.

### PL and ODMR set-ups

For VV^0^, the sample is excited with a 976 nm diode laser (40 mW at sample, focused with a ×50 IR objective) and PL is measured with an InGaAs detector (1000–1300 nm after filtering). For ${\rm{V}}_{{\rm{Si}}}^ -$, the sample is excited with a 780 nm diode laser (10 mW at sample) and PL is measured with a Si detector (850–950 nm after filtering, allowing simultaneous VV^0^ PL recording). Simplified schematics for the PL/ODMR set-ups are given in Supplementary Fig. 1. All given optical powers were measured at the sample. Excitation spectra are recorded by inserting a monochromator immediately after a 100 W Xe white light source in the optical set-up. Emission spectra or measurements at selective zero-phonon lines (ZPL) are recorded by inserting a monochromator before the detector. For the wavelength dependence, a set of laser diodes were successively collimated into a 300 μm multi-mode fiber and re-emitted into free space so as to ensure a constant spot position on the sample. It should also be noted there is no significant PL contribution from 405 or 365 nm illumination alone.

### Transients and modeling

The three-pulse scheme used for the photo-dynamics requires careful choice of the 976 nm measurement pulse duration (0.1 ms) as it is necessary for exciting PL but can also change the charge state of VV. A long pulse would effectively smooth the decays and prevent good fitting at short times. The experimental decay rates and steady states are obtained from fitting with a stretched exponential function, with separate fitting parameters for each power, wavelength and temperature dependence. The actual decay curves are shown in Supplementary Fig. 3, and all simulated lines in Fig. 3 are from the rate-equation model. All details on this model are given in Supplementary Note 1, regarding e.g., simulation of the wavelength dependence (Grimmeiss model for deep trap^48^) and the exact rate equations.

In total, 12 parameters are used for a simultaneous fit over a set of 70 decays curves, with the simulation results shown by the lines in Fig. 3a, b, e. Looking at the decays in Fig. 3a, the fits are in excellent agreement in certain ranges (833 nm pumping) but do not account for all the charge dynamics as seen in the left figures. For 365 nm pumping, electron-hole pair generation dominates over all photoionization processes, and the free carrier concentration is determined by the recombination with all involved traps, not just VV. Hence for such a simple model, large discrepancies are expected. In addition, the simulation strictly considers a single defect while measuring an ensemble can easily smooth features in the decays, e.g., due to local variations in strain, charge, light intensity, etc.

### Electron spin resonance

ESR experiments were realized on a X-band (dielectric resonator, 5 mm internal diameter) ELEXSYS E580 Bruker spectrometer at 15 K. In the differential experiments presented in Fig. 4, one important issue is the simultaneous effect of illumination on both spin (polarization, relaxation) and charge properties, and hence on the ESR intensity. The results presented may convolute both aspects, unlike the PL experiments which are clearly related to the charge state. Turning the lasers on and off would avoid this concern, however the signal was then simply too weak to obtain any information on N, V_C_, or V_Si_. The 940 and 976 nm excitation lasers are sufficiently close in energy to limit most effects but those related to the sharp photoionization transition in VV. In addition, these wavelengths are both in the VV absorption sideband, but close enough to see no appreciable differences in spin polarization due to inter-system crossing mechanisms. Similarly, they are also above the longest ZPL wavelength of ${\rm{V}}_{{\rm{Si}}}^ -$ (917 nm^5^), preventing any spin polarization.

### Data availability

The data that support the findings of this study are available upon request to the corresponding author.

## Electronic supplementary material

Supplementary Information

## References

[1] Waldherr G (2014). Quantum error correction in a solid-state hybrid spin register. Nature.

[2] Maze JR (2008). Nanoscale magnetic sensing with an individual electronic spin in diamond. Nature.

[3] Toyli DM, de las Casas CF, Christle DJ, Dobrovitski VV, Awschalom DD (2013). Fluorescence thermometry enhanced by the quantum coherence of single spins in diamond. Proc. Natl Acad. Sci. USA.

[4] Kucsko G (2013). Nanometre-scale thermometry in a living cell. Nature.

[5] Baranov PG (2011). Silicon vacancy in SiC as a promising quantum system for single-defect and single-photon spectroscopy. Phys. Rev. B.

[6] Koehl WF, Buckley BB, Heremans FJ, Calusine G, Awschalom DD (2011). Room temperature coherent control of defect spin qubits in silicon carbide. Nature.

[7] Kraus H (2013). Room-temperature quantum microwave emitters based on spin defects in silicon carbide. Nat. Phys..

[8] Christle DJ (2014). Isolated electron spins in silicon carbide with millisecond coherence times. Nat. Mater..

[9] Widmann M (2014). Coherent control of single spins in silicon carbide at room temperature. Nat. Mater..

[10] Christle DJ (2017). Isolated spin qubits in sic with a high-fidelity infrared spin-to-photoninterface. Phys. Rev. X.

[11] Janzén E (2009). The silicon vacancy in SiC. Phys. B: Condens. Matter.

[12] Torpo L, Staab T, Nieminen R (2002). Divacancy in 3C- and 4H-SiC: an extremely stable defect. Phys. Rev. B.

[13] Son NT (2006). Divacancy in 4H-SiC. Phys. Rev. Lett..

[14] Falk AL (2013). Polytype control of spin qubits in silicon carbide. Nat. Commun..

[15] Booker ID (2014). Carrier lifetime controlling defects Z 1/2 and RB1 in standard and chlorinated chemistry grown 4H-SiC. Cryst. Growth Design.

[16] Booker ID (2016). Donor and double-donor transitions of the carbon vacancy related EH 67 deep level in 4H-SiC. J. Appl. Phys..

[17] Matsumoto T, Poluektov OG, Schmidt J, Mokhov EN, Baranov PG (1997). Electronic structure of the shallow boron acceptor in 6H-SiC:mA pulsed EPR/ENDOR study at 95 GHz. Phys. Rev. B.

[18] Isoya J (2008). EPR identification of intrinsic defects in SiC. Phys. Status Solidi (b).

[19] Gali A (2012). Excitation spectrum of point defects in semiconductors studied by time-dependent density functional theory. J. Mater. Res..

[20] Gordon L, Janotti A, Van de Walle CG (2015). Defects as qubits in 3C and 4HSiC. Phys. Rev. B.

[21] Weber JR (2011). Defects in SiC for quantum computing. J. Appl. Phys..

[22] Umeda T (2005). EPR and theoretical studies of negatively charged carbon vacancy in 4HSiC. Phys. Rev. B.

[23] Son NT (2012). Negative-system of carbon vacancy in 4H-SiC. Phys. Rev. Lett..

[24] Umeda T, Morishita N, Ohshima T, Itoh H, Isoya J (2009). Photo-EPR study of vacancy-type defects in irradiated n-type 4H-SiC. Mater. Sci. Forum.

[25] Aslam N, Waldherr G, Neumann P, Jelezko F, Wrachtrup J (2013). Photo-induced ionization dynamics of the nitrogen vacancy defect in diamond investigated by single-shot charge state detection. New. J. Phys..

[26] Siyushev P (2013). Optically controlled switching of the charge state of a single nitrogen-vacancy center in diamond at cryogenic temperatures. Phys. Rev. Lett..

[27] Bassett LC, Heremans FJ, Yale CG, Buckley BB, Awschalom DD (2011). Electrical tuning of single nitrogen-vacancy center optical transitions enhanced by photoinduced fields. Phys. Rev. Lett..

[28] Dhomkar S, Henshaw J, Jayakumar H, Meriles CA (2016). Long-term data storage in diamond. Sci. Adv..

[29] Han KY, Kim SK, Eggeling C, Hell SW (2010). Metastable dark states enable ground state depletion microscopy of nitrogen vacancy centers in diamond with diffraction-unlimited resolution. Nano Lett..

[30] Chen X (2015). Subdiffraction optical manipulation of the charge state of nitrogen vacancy center in diamond. Light: Sci. Appl..

[31] Doherty MW (2016). Towards a room-temperature spin quantum bus in diamond via electron photoionization, transport, and capture. Phys. Rev. X.

[32] Jenny JR (2004). Development of large diameter high-purity semi-insulating 4H-SiC wafers for microwave devices. Mater. Sci. Forum.

[33] Zargaleh SA (2016). Evidence for near-infrared photoluminescence of nitrogen vacancy centers in 4H-SiC. Phys. Rev. B.

[34] Seo H (2016). Quantum decoherence dynamics of divacancy spins in silicon carbide. Nat. Commun..

[35] Tyryshkin AM (2011). Electron spin coherence exceeding seconds in high-purity silicon. Nat. Mater..

[36] Csóré A, von Bardeleben HJ, Cantin JL, Gali A (2017). Characterization and formation of NV centers in 3C, 4H and 6H SiC: An ab initio study. Phys. Rev. B.

[37] Golter DA, Lai CW (2017). Optical switching of defect charge states in 4H-SiC. Sci. Rep.

[38] Gerstmann U, Rauls E, Frauenheim T, Overhof H (2003). Formation and annealing of nitrogen-related complexes in SiC. Phys. Rev. B.

[39] Zolnai Z, Son NT, Hallin C, Janzén E (2004). Annealing behavior of the carbon vacancy in electron-irradiated 4H-SiC. J. Appl. Phys..

[40] Schmid F (2006). Deactivation of nitrogen donors in silicon carbide. Phys. Rev. B.

[41] Carlos WE, Garces NY, Glaser ER, Fanton MA (2006). Annealing of multivacancy defects in 4HSiC. Phys. Rev. B.

[42] Embley JS (2017). Electron spin coherence of silicon vacancies in proton-irradiated 4 H -SiC. Phys. Rev. B.

[43] Hornos T, Gali A, Svensson BG (2011). Large-scale electronic structure calculations of vacancies in 4H-SiC using the heyd-scuseria-ernzerhof screened hybrid density functional. Mater. Sci. Forum.

[44] Fu KMCMC, Santori C, Barclay PE, Beausoleil RG (2010). Conversion of neutral nitrogen-vacancy centers to negatively charged nitrogen-vacancy centers through selective oxidation. Appl. Phys. Lett..

[45] Fuchs F (2015). Engineering near-infrared single-photon emitters with optically active spins in ultrapure silicon carbide. Nat. Commun..

[46] Son NT, Carlsson P, ul Hassan J, Magnusson B, Janzén E (2007). Defects and carrier compensation in semi-insulating 4HSiC substrates. Phys. Rev. B.

[47] Chandrashekhar MVS (2012). High purity semi-insulating 4H-SiC epitaxial layers by defect-competition epitaxy: controlling Si vacancies. Appl. Phys. Express.

[48] Grimmeiss HG, Ledebo LA (1975). Photo-ionization of deep impurity levels in semiconductors with non-parabolic bands. J. Phys. C: Solid State Phys..

[49] Galeckas A, Grivickas P, Grivickas V, Bikbajevas V, Linnros J (2002). Temperature dependence of the absorption coefficient in 4H- and 6H-silicon carbide at 355 nm laser Pumping wavelength. Phys. Status Solidi (a).

[50] Morello A (2010). Single-shot readout of an electron spin in silicon. Nature.

