# Supplementary material for: Optical charge state control of spin defects in 4H-SiC
Source: Nat Commun. 2017 Nov 30;8:1876. doi: 10.1038/s41467-017-01993-4 (PMC5709515; doi:10.1038/s41467-017-01993-4)
Supplement: Supplementary file 1 — Supplementary Information [file 41467_2017_1993_MOESM1_ESM.pdf]

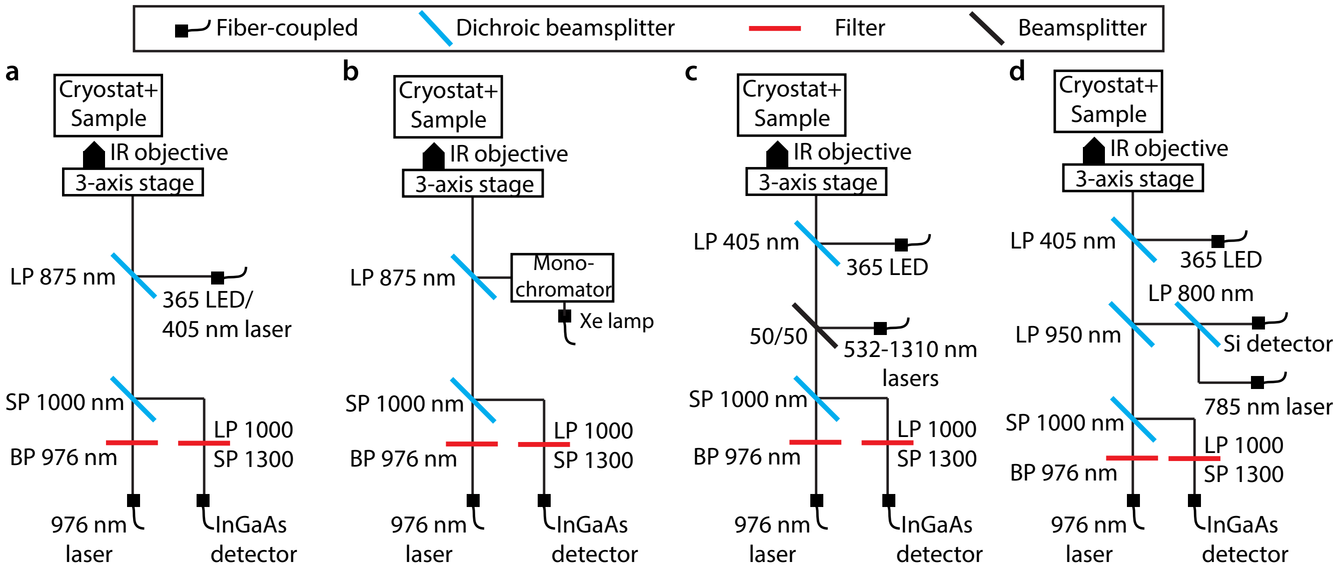

FIG. 1. *

Supplementary Figure 1. Schematic of the PL and ODMR setups for the various pumping experiments. SP stands for short-pass and LP stands for long-pass. **(a)** VV setup with 976 nm and 365 or 405 nm excitation. **(b)** VV setup for excitation dependence using white light source. **(c)** VV setup for three-pulse experiments. **(d)** Combined VV and V_{Si} setup with 780 nm excitation.

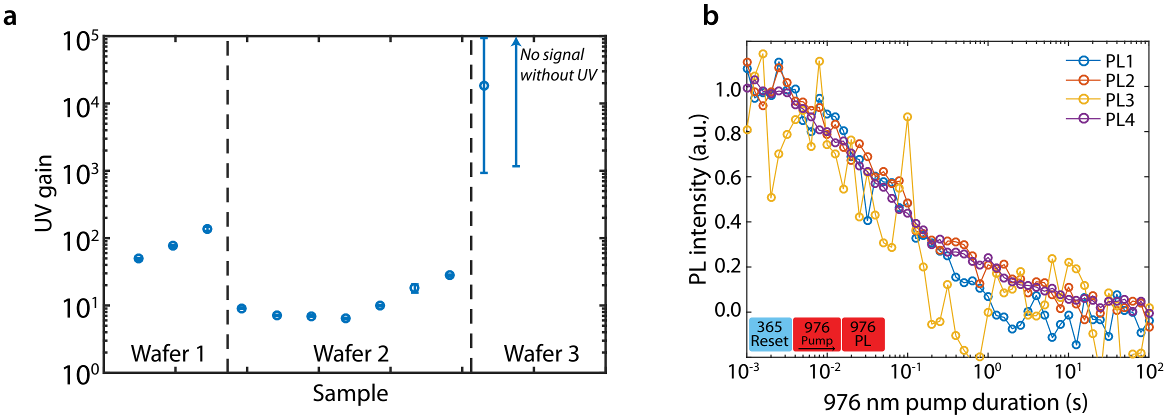

FIG. 2. *

Supplementary Figure 2. Charge conversion efficiency in different VV defect types and samples. (a) Scatter plot of the UV (365 nm) enhancement measured from PL2 ODMR at different locations in 3 separate high-purity semi-insulating wafers. The variation in UV gain reflects different local impurity concentrations. For wafer 3 (right), the signal without UV is below our detection limit and only a lower bound for the gain is given. The errors bars are 95% confidence interval from fit of the PL2 ODMR spectrum. **(b)** Transient decays under 976 nm pumping and measured for each of the VV defect types at 6 K. Each of the PL contributions are separated using a monochromator set at each of the defect zero-phonon line wavelengths. The decays do not significantly vary between the various VV defect types: this allowed us to directly integrate over all the PL1 to PL4 signals in the experiments of the main text. This was necessary in order to obtain sufficient signal-to-noise ratio (in particular above 100 K) since averaging was strongly hindered by the long timescales of the decays.

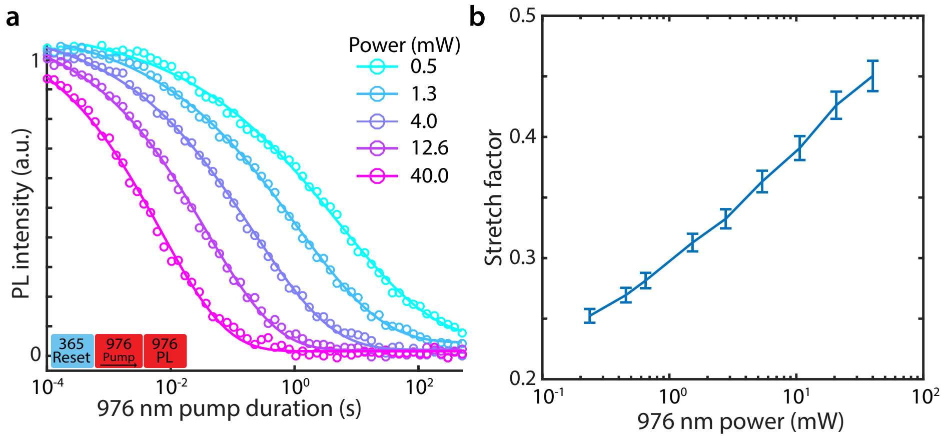

FIG. 3. *

Supplementary Figure 3. Stretched exponential fitting. (a) Transient decay from 976 nm pumping and 365 nm reset. The lines are from a stretch exponential fit and reproduce well the decay behavior. **(b)** Stretch factor n from the fitting in (a) as a function of 976 nm laser power. The errors bars are 95% confidence interval from the decay fit.

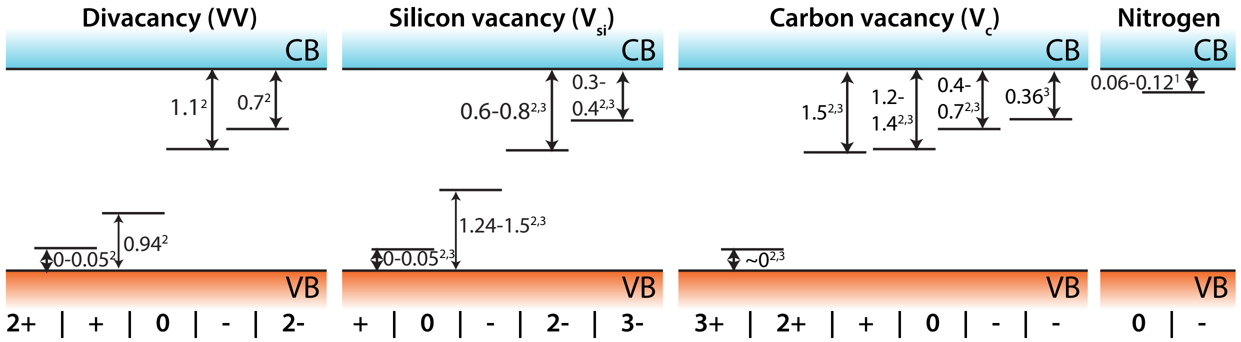

FIG. 4. *

Supplementary Figure 4. Transition levels of relevant impurities in semi-insulating in 4H-SiC. Reproduced from [1] (1), [2] (2) (calculated bandgap $E_g = 3.17$ eV) and [3] (3) (calculated bandgap $E_g = 3.16$ eV). Each transition state is between the two charge configurations as given by the numbers at the bottom of the figure. The transition energies correspond to the Fermi level position such that for lower energies the defect is stable in the charge state q, and for higher energies is stable in the charge state $q - 1$.

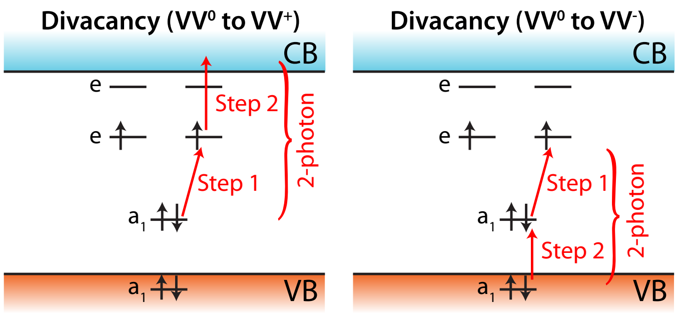

FIG. 5. *

Supplementary Figure 5. Charge conversion in the single-particle model. Charge conversion from VV^0 to VV^+ (left) or VV^- (right) by two-photon absorption shown using the VV^0 single-particle electronic structure [2]. VV^0 can absorb one photon to reach its excited state (one electron from the highest a_1 state to the lower e state), and a second photon to ionize either to VV^+ (one electron from the lower e state to the conduction band) or to VV^- (one electron from the valence band to the highest a_1 state).

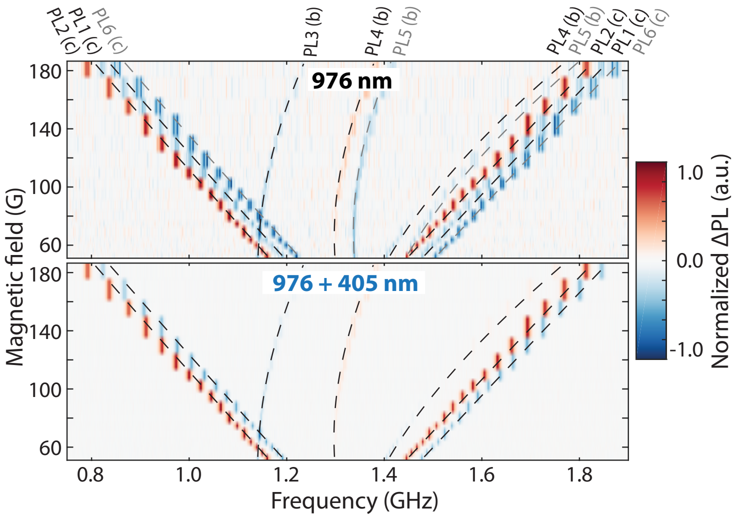

FIG. 6. *

Supplementary Figure 6. Magnetic field dependence of CW-ODMR spectrum at 6 K. Identification of the VV defect type resonances by simultaneous microwave frequency and magnetic field sweeping. Top: 976 nm excitation only, bottom: 976 nm and 405 nm excitation. The ODMR intensity is normalized to emphasize which defect is enhanced. Following results from PL only (Figure 1 of the main text), the PL1 to PL4 defects (*hh*, *kk*, *hk* and *kh* sites) are enhanced by the UV (405 nm) excitation, while PL5 and PL6 do not change.

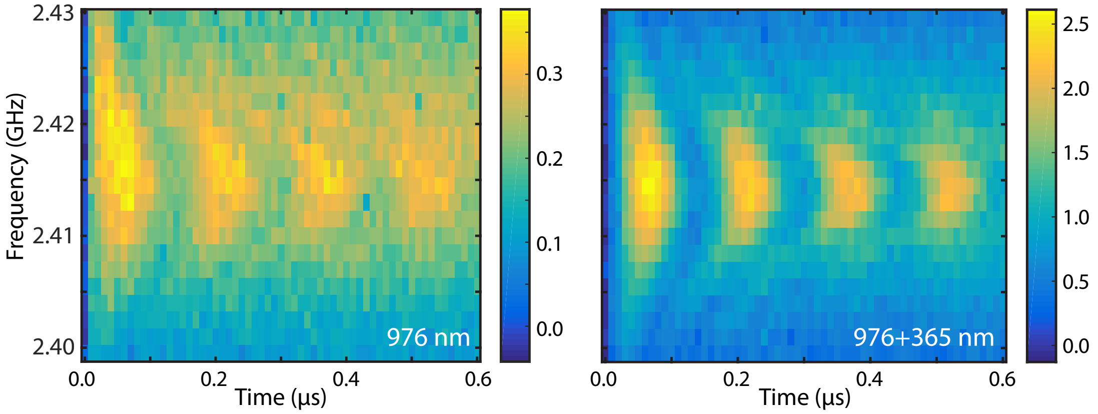

FIG. 7. *

Supplementary Figure 7. Rabi oscillations in implanted sample at 6 K. PL2 ODMR measurements realized at 2.404 GHz (∼ 400 G) in the VV implanted layer peak as shown in Figure 2(c) of the main text. With 976 nm illumination only (left), the low Rabi contrast (45 %) is mainly from the overlapping bulk ODMR. When 365 nm is also turned on (right), the contrast increases to 67 % as the implanted ODMR signal with less inhomogeneous broadening dominates over the bulk.

SUPPLEMENTARY NOTE 1 - TRANSIENT FITS AND MODELING

In Figure 3(a) of the main text, typical decay rates from light excitation were shown to have a stretched exponential function $A\exp(-(Rt)^n)$ with amplitude A, rate R and stretch factor n. Such form is fairly common in relaxation/recombination experiments, arising from either statistical distributions (decay rates, activation energies) or due to trapping mechanisms and the non-linearity of recombination processes [4, 5]. This second explanation is more likely in our system, though the ensemble measurement, both spatially and spectrally (PL signal is integrated over PL1-4), likely provides some small distribution of relevant rates in the system.

In Supplementary Figure 3, the stretch exponential fit (in (a)) is shown for the case of 976 nm pumping after reset with 365 nm excitation, with the corresponding fitted stretch factors (in (b)). The fits are excellent with very low stretch factors (0.25-0.45) corresponding to highly non-exponential decays. For 976 nm pumping, the stretch factors are laser power dependent contrary to other wavelengths used (not shown here) where the stretch factor is nearly constant. This dependence with 976 nm illumination likely reflects the balance between VV hole photoionization, immediate hole re-capture and electron photo-emission from traps followed by capture by VV.

We now turn toward the rate equation modeling of the transients using the model shown in Figure 3(d) of the main text. This model has to be largely simplified to avoid over-parametrization as there are very few known parameters available in literature for the divacancies, specifically regarding cross-sections and ionization/activation energies. The model is therefore restricted to: two charge states for the divacancy, one with electron capture/photoemission from/to the conduction band and the other with hole capture/photoemission (two-photon) from/to the valence band, along with two charge states for an unknown trap to which charges are transferred during photoemission. In addition, electron photoemission from the trap is allowed to reflect possible ionization of nitrogen shallow donors and carbon vacancies. From a divacancy point of view, only the free carrier concentration is directly relevant, and surrounding defect species are hard to distinguish. The modeled trap hence does not necessarily reflect a single defect, only important processes. A electron-hole thermal generation is also added to simulate the temperature dependence: this choice was initially given by the simulation as other temperature dependences did not provide a good fit to the transients. This mechanism may originates from thermal emission of shallow defects, capture barriers or change in carrier lifetimes. Finally, electron-hole generation is allowed for above-bandgap excitation.

The rate equations for the divacancy (VV), trap (T) and free carrier (electrons n and holes p) populations and charge states (ρ) at a given temperature T, excitation energy E and power P is as follows:

$$\frac{d\rho_{\mathrm{VV}^0}}{dt} = -R_{\mathrm{ncv}}v_{\mathrm{nth}}(T)n\rho_{\mathrm{VV}^0} + R_{\mathrm{pcv}}v_{\mathrm{pth}}(T)p\rho_{\mathrm{VV}^-} + R_{\mathrm{nev}}(E)N_{\mathrm{ph}}(P,E)\rho_{\mathrm{VV}^-} - R_{\mathrm{pev}}(E)N_{\mathrm{ph}}^2(P,E)\rho_{\mathrm{VV}^0} \tag{1}$$

$$\frac{dn}{dt} = G_{\mathrm{np}}(P,E,T) - R_{\mathrm{ncv}}v_{\mathrm{nth}}(T)n\rho_{\mathrm{VV}^0} + R_{\mathrm{nev}}(E)N_{\mathrm{ph}}(P,E)\rho_{\mathrm{VV}^-} - R_{\mathrm{nct}}v_{\mathrm{nth}}(T)n\rho_{\mathrm{T}^0} + R_{\mathrm{net}}(E)N_{\mathrm{ph}}(P,E)\rho_{\mathrm{T}^-} \tag{2}$$

$$\frac{dp}{dt} = G_{\mathrm{np}}(P,E,T) - R_{\mathrm{pcv}}v_{\mathrm{pth}}(T)p\rho_{\mathrm{VV}^-} + R_{\mathrm{pev}}(E)N_{\mathrm{ph}}(P,E)\rho_{\mathrm{VV}^0} - R_{\mathrm{pct}}v_{\mathrm{pth}}(T)p\rho_{\mathrm{T}^-} \tag{3}$$

$$Q = n - p + \rho_{\mathrm{VV}^-} + \rho_{\mathrm{T}^-} \tag{4}$$

$$\rho_{\mathrm{VV}^-} = V_{\mathrm{pop}} - \rho_{\mathrm{VV}^0} \tag{5}$$

$$\rho_{\mathrm{T}^-} = T_{\mathrm{pop}} - \rho_{\mathrm{T}^0} \tag{6}$$

and with

$$N_{\mathrm{v}}(T) = 2(2\pi m_{h,\mathrm{dos}}k_{\mathrm{B}}T/h^2)^{3/2} \tag{7}$$

$$v_{\mathrm{nth}}(T) = \sqrt{3k_{\mathrm{B}}T/m_{e,\mathrm{cond}}} \text{ and } \mathrm{v_{pth}(T)} = \sqrt{\mathrm{3k_B T/m_{h,cond}}} \tag{8}$$

$$N_{\mathrm{ph}}(P,E) = P/(A_{\mathrm{spot}}(E)E) \tag{9}$$

$$G_{\mathrm{np}}(P,T) = N_{\mathrm{ph}}(P,E)\alpha(E,T) + G_{\mathrm{np,thermal}}\exp\left(\frac{-E_{\mathrm{a}}}{k_{\mathrm{B}}T}\right) \tag{10}$$

$$\alpha(E,T) = \alpha_0\left(\frac{(E - E_{\mathrm{g}}(T) - E_{\mathrm{ph}})^2}{1 - \exp(-E_{\mathrm{ph}}/k_{\mathrm{B}}T)} + \frac{(E - E_{\mathrm{g}}(T) + E_{\mathrm{ph}})^2}{\exp(E_{\mathrm{ph}}/k_{\mathrm{B}}T) - 1}\right) \tag{11}$$

The coefficients (rates or cross-sections) $R_{\eta\beta\delta}$ are designated by three parameters: $\eta = n, p$ if the conduction (electrons) or valence (holes) bands are involved, $\beta = c, e$ for capture or emission processes, and $\delta = v, t$ for divacancies or traps. For simplicity, all the rates were supposed temperature-independent. G_{np} is the electron-hole generation

process with the absorption coefficient α ($\alpha_0 = 6200\ \mathrm{cm}^{-1}\mathrm{eV}^{-2}$) for 4H-SiC taken from [6], depending on a temperature dependent bandgap value (follows the Rashni formula as given in [6]) and the longitudinal phonon energy $E_{\mathrm{ph}} =$ 70 meV. Our samples are all 500 μm thick, hence there is no significant decay of the optical power due to absorption at all measured wavelengths. N_{v} is the conduction band density of states. v_{nth} and v_{pth} are the conduction and valence band thermal velocities. N_{ph} is the photon number per area. Q is the total charge of the system and links the divacancy and free carrier charge configuration to the trap state due to charge conservation. V_{pop} and T_{pop} are the total divacancy and trap populations independent of their charge state. V_{pop} is normalized to 1 and all populations as well as capture rates are given as a ratio to the VV population. For simplicity, T_{pop} is also set to 1 though this directly impacts the corresponding trap rates (leaving T_{pop} as a free parameter prevents good fitting due to over-parametrization). E_{a} is the activation energy for thermal electron-hole generation with rate $G_{\mathrm{np,thermal}}$ at $T \to \infty$. A_{spot} is the laser spot size which was estimated for each laser diode taking into account all the optics in the setup. Though it was not done here, averaging over the depth profile of A_{spot} could provide a better fit to the data. k_{B} is the Boltzmann constant, m_0 is the free electron mass, $m_{e,\mathrm{cond}} = 0.37m_0$ and $m_{h,\mathrm{cond}} = 0.83m_0$ are the effective electron and hole mass for conductivity calculations in 4H-SiC, $m_{e,\mathrm{dos}} = 0.77m_0$ and $m_{h,\mathrm{dos}} = 0.91m_0$ are the effective electron and hole mass for density of state calculations in 4H-SiC [7, 8].

The photoionization rates $R_{\mathrm{net}}(E)$, $R_{\mathrm{nev}}(E)$, $R_{\mathrm{pev}}(E)$ are wavelength dependent and would normally require one fitting parameter for each wavelength used. For simplicity, we use the model first developed by Lucovsky [9] for deep impurities and later improved by Grimmeiss [10]. Those models roughly approximate the defect ion core potential as a delta function, and give the following functional form for the photoionization rates:

$$R_{\mathrm{net}}(E) = R^0_{\mathrm{net}} \frac{(E - E_{\mathrm{net}})^{3/2}}{E\left(E + E_{\mathrm{net}}\left(m_0/m_{e,\mathrm{dos}} - 1\right)\right)^2} \tag{12}$$

$$R_{\mathrm{nev}}(E) = R^0_{\mathrm{nev}} \frac{(E - E_{\mathrm{nev}})^{3/2}}{E\left(E + E_{\mathrm{nev}}\left(m_0/m_{e,\mathrm{dos}} - 1\right)\right)^2} \tag{13}$$

$$R_{\mathrm{pev}}(E) \approx R^0_{\mathrm{pev}} \frac{(E - E_{\mathrm{pev}})^{3/2}}{E\left(E + E_{\mathrm{pev}}\left(m_0/m_{h,\mathrm{dos}} - 1\right)\right)^2} f(E_{\mathrm{ZPL}}, \Delta E_{\mathrm{PSB}}) \tag{14}$$

with E_{nev} and E_{pev} the electron and hole photoionization energies for VV^- and VV^0 respectively. E_{net} here is a characteristic electron photoionization energy for the traps, though the actual wavelength dependence is likely much more complicated. For R_{pev}, the transition involves two-photon absorption, first exciting VV^0 from ground state to excited state followed by hole emission to the valence band. The wavelength dependence of the first step is captured in $f(E_{\mathrm{ZPL}}, \Delta E_{\mathrm{PSB}})$, were f is the Poisson distribution (in continuous form), $E_{\mathrm{ZPL}} \approx 1.1$ eV and $\Delta E_{\mathrm{PSB}} \approx 0.25$ eV are respectively the zero-phonon line transition energy (ZPL) and energy difference between ZPL and the maximum of the phonon sideband (obtained from [11]). Because f varies much faster with wavelength than the Grimmeiss function, we neglected the latter in the simulation to minimize the number of parameters.

In order to simulate the three-pulse (reset/pump/measure) experiments, the reset pulse and corresponding initial populations are taken from the steady state solution of the rate equations under pumping at the reset wavelength. Between the pump and measure, there is a typical 0.1 ms delay in the experiments without any illumination, and we checked that the simulated divacancy and trap charge populations did not change between this interval.

SUPPLEMENTARY NOTE 2 - ODMR MEASUREMENTS

Optically detected magnetic resonance (ODMR) experiments are all realized in a home-built confocal microscope setup, and the PL measured via lock-in detection methods at a typical frequency of $\approx$ 400 Hz. In continuous-wave (CW) experiments, the microwave is switched on and off at the lock-in frequency, and in pulsed experiments, the sequences are repeated at the lock-in rate. In Supplementary Figure 6, a CW-ODMR spectrum as a function of both microwave frequency and static magnetic field is shown. The magnetic field is created using a neodymium disc (76 mm diameter) magnet on a linear stage. Each defect sites for VV are simulated according to the g-factors as well as zero-field splitting parameters D and E given in [12] (Supplementary Material).

All the defects from PL1 to PL6 are observed with 976 nm excitation, and following the PL spectrum measured in the Figure 1 of the main text, only PL1 to PL4 are enhanced when 405 nm is also turned on. In Supplementary Figure 6 (bottom), it is hard to resolve PL5 and PL6 as their intensities, which are constant under 405 nm illumination, appear negligible compared to the enhanced signal of the other defects.

In Figure 2(c) of the main text, the ODMR spectrum for a 500 nm high defect density layer created by high-energy carbon implant was shown to be only visible with UV illumination. In Supplementary Figure 7, Rabi oscillations were measured at the resonant frequency of the defect layer: without UV (365 nm) excitation, the ODMR signal is dominated by the bulk contribution with a larger inhomogeneous broadening ($\approx$ 20 MHz) and therefore lower Rabi contrast (45 %, defined as the relative amplitude between the $\pi/2$ and π rotations). With UV, the layer signal dominates and the Rabi contrast increases (67 %) due to depth confinement and reduced inhomogeneous broadening ($\approx$ 2 MHz). The ODMR intensity is also nearly 10 times larger in this case.

SUPPLEMENTARY REFERENCES

[1] Ivanov, I. G., Henry, A. & Janzén, E. Ionization energies of phosphorus and nitrogen donors and aluminum acceptors in 4H silicon carbide from the donor-acceptor pair emission. *Physical Review B* **71**, 241201 (2005).

[2] Gordon, L., Janotti, A. & Van de Walle, C. G. Defects as qubits in 3C and 4HSiC. *Physical Review B* **92**, 045208 (2015).

[3] Hornos, T., Gali, A. & Svensson, B. G. Large-Scale Electronic Structure Calculations of Vacancies in 4H-SiC Using the Heyd-Scuseria-Ernzerhof Screened Hybrid Density Functional. *Materials Science Forum* **679-680**, 261–264 (2011).

[4] Van de Walle, C. G. Stretched-exponential relaxation modeled without invoking statistical distributions. *Physical Review B* **53**, 11292–11295 (1996).

[5] Chen, R. Apparent stretched-exponential luminescence decay in crystalline solids. *Journal of Luminescence* **102-103**, 510–518 (2003).

[6] Galeckas, A., Grivickas, P., Grivickas, V., Bikbajevas, V. & Linnros, J. Temperature Dependence of the Absorption Coefficient in 4H- and 6H-Silicon Carbide at 355 nm Laser Pumping Wavelength. *physica status solidi (a)* **191**, 613–620 (2002).

[7] Son, N. T. *et al.* Electron effective masses in 4H SiC. *Applied Physics Letters* **66**, 1074–1076 (1995).

[8] Son, N. T. *et al.* Hole effective masses in 4H SiC. *Physical Review B* **61**, R10544–R10546 (2000).

[9] Lucovsky, G. On the photoionization of deep impurity centers in semiconductors. *Solid State Communications* **88**, 879–882 (1993).

[10] Grimmeiss, H. G. & Ledebo, L. A. Photo-ionization of deep impurity levels in semiconductors with non-parabolic bands. *Journal of Physics C: Solid State Physics* **8**, 2615–2626 (1975).

[11] Golter, D. A. & Lai, C. W. Optical switching of defect charge states in 4H-SiC. Preprint at http://arxiv.org/abs/1707.01558 (2017).

[12] Falk, A. L. *et al.* Polytype control of spin qubits in silicon carbide. *Nature Communications* **4**, 1819 (2013).
